# Chronic malaria and hyper-reactive malarial splenomegaly: a retrospective study on the largest series observed in a non-endemic country

**DOI:** 10.1186/s12936-016-1274-x

**Published:** 2016-04-21

**Authors:** Zeno Bisoffi, Stefania Leoni, Andrea Angheben, Anna Beltrame, Franklyn Esoka Eseme, Federico Gobbi, Claudia Lodesani, Stefania Marocco, Dora Buonfrate

**Affiliations:** Centre for Tropical Diseases, Sacro Cuore – Don Calabria Hospital, 37024 Negrar, Verona Italy; Ospedale Dell’Angelo, Via Don Federico Tosatto, 147, 30174 Venezia Mestre, Italy; Medici Senza Frontiere Italia, Via Magenta 5, 00186 Rome, Italy

## Abstract

**Background:**

Chronic malaria is usually defined as a long-term malarial infection in semi-immune subjects, usually without fever or other acute symptoms. The untreated infection may evolve to hyper-reactive malarial splenomegaly (HMS), a life-threatening complication. This paper describes the largest series of HMS ever observed outside endemic countries, and the clinical outcome after a single anti-malarial treatment. Contrarily to most authors, still reporting the traditional, long-term treatment, regardless possible further exposure, the patients in this series did not receive any further prophylaxis if they were not re-exposed to malaria infection.

**Methods:**

A retrospective, longitudinal study, describing all patients with HMS diagnosed at the Centre for Tropical Diseases of Negrar, Verona, took place over a 25-year period. HMS was defined by a longitudinal spleen diameter ≥16 cm, IgM ≥ 2.5 g/L, anti-malarial antibody titre ≥160, exclusion of other causes of splenomegaly. The short-term (≤6 months) clinical outcome after a single anti-malarial treatment was analysed and so was the long-term outcome of subjects re-exposed to malaria and submitted or not to anti-malarial prophylaxis or intermittent treatment. The association of the outcome with the main independent variables was first assessed with univariate analysis. Logistic regression was also performed.

**Results:**

Forty-four subjects with HMS were retrieved. Of those with a short-term follow-up visit (<6 months, median 43 days) available before returning to endemic areas, 20/22 resulted improved/cured, two were unchanged. Of 22 expatriates seen at long-term follow-up after re-exposure, 18 were improved/cured, including eight out of nine who had followed an anti-malarial prophylaxis and 10/13 who had opted for the alternative of an intermittent treatment.

**Conclusion:**

HMS is the most severe form of chronic malaria. A single anti-malarial treatment is probably adequate to treat HMS in the absence of re-exposure, while an adequate prophylaxis is necessary for patients exposed again to malaria transmission. Intermittent treatment would probably be the only viable approach in endemic countries.

## Background

Chronic malaria is a long-term infection in semi-immune subjects. It is usually characterized by the absence of fever or any other acute symptoms, so that this condition has long been defined as asymptomatic carriage of malaria parasites [1]. However, it has been argued recently that asymptomatic malaria does not exist [2], as the chronic presence of malaria parasites is a cause of anaemia, predisposes to other infections, and is a common cause of maternal complications, to cite only some of the problems related to chronic malaria carriage.

Hyper-reactive malarial splenomegaly (HMS) is probably the most severe form of chronic malaria [3]. The main characteristics of the syndrome have been summarized in a recent review [4]. Overwhelming infections are a major cause of death in these patients, and the syndrome is characterized by high mortality if not properly treated. Malaria microscopy is often negative in these patients, leading researchers to speculate that the progression of the syndrome is not related to the presence of malaria infection in blood, but rather to an immune-mediated mechanism. However, it has been observed recently that more sensitive diagnostic methods, such as quantitative buffy coat (QBC), rapid diagnostic tests (RDTs) or polymerase chain reaction (PCR), may yield a positive result in microscopically negative patients [4, 5]. Moreover, a single anti-malarial treatment has been able to cure the syndrome according to a few anecdotal reports [6]. In addition, long-term chloroquine prophylaxis, historically the mainstay of treatment of HMS by virtue of its immune-modulating properties [7], has apparently become less effective in recent years, in face of widespread resistance by *Plasmodium falciparum* to this molecule.

A previous paper [8] described the characteristics of ‘early HMS’ (e-HMS), a condition that was not previously considered, as it does not fulfill the case definition criteria of HMS, but that is at risk of evolving into ‘full-blown HMS’ if left untreated. This paper describes the main characteristics of all patients with full-blown HMS (mostly Italian expatriates, but also immigrants) seen in the last 25 years at the Centre for Tropical Diseases (CTD) of Negrar, Verona, Italy.

The main objectives of the present study were: (a) to describe the main clinical and laboratory findings of immigrants and expatriates diagnosed at CTD with HMS; (b) to analyse the short-term and long-term outcome of HMS patients after a single anti-malarial treatment, followed by long-term prophylaxis (or by intermittent treatment), for those who returned to endemic areas for malaria.

## Methods

### Study design

#### Retrospective-longitudinal study

The clinical records of patients were retrieved as described previously [8], covering the period from 1 January, 1990 to 3 September, 2015 (date of the last follow-up visit of the last patient). Briefly, patients with splenomegaly or with raised IgM and with anti-malarial antibody titre ≥160 (IFAT-Biomérieux) were retrieved from the CTD patient database. Patients meeting the case definition of full-blown HMS as outlined below were included, while the remaining subjects, meeting the tentative case definition of e-HMS (spleen diameter <16 cm and/or normal IgM), that were the object of the previous study, were excluded. Patients were then traced prospectively for any further visit short term (≤6 months) or long term (>6 months).

The following case definition for HMS was used:

- antimalarial antibody titre (IFAT-Biomérieux) ≥1/160, PLUS:
- massive splenomegaly (ultrasound longitudinal diameter, corresponding to spleen length, ≥16 cm) (normal value in healthy adults 8–11 cm) [9];
- IgM level ≥2 SD above the local mean (or ≥2.5 g/L according to the local laboratory);
- no other identifiable cause of splenomegaly or of raised IgM, such as schistosomiasis, cirrhosis, haematological conditions, and others as described previously [8].

Final outcome was assessed on the basis of the last follow up visit.

#### Data entry and data elaboration

Data were exported to a pre-structured Excel file, including the main epidemiological, clinical and laboratory findings as well as the results of an ultrasound scan, as available at baseline (T1), short-term follow-up (T2) and long-term follow-up (T3) [8].

#### Definition of follow-up criteria

- Cured: absence of splenomegaly and normal IgM value;
- Improved: ≥1 cm decrease of the spleen diameter, or unchanged spleen diameter and ≥1 g/dL decrease of IgM level;
- Unchanged: <1 cm or no variation of the spleen diameter and <1 g/dL or no variation of IgM level;
- Progressed: ≥1 cm increase of the spleen diameter, or unchanged spleen diameter and ≥1 g/dL increase of IgM level.

#### Laboratory methods for malaria detection

Thick and thin film microscopy, as well as the QBC Malaria Test (QBC Diagnostics Inc, Philipsburg, USA), were performed following the routine procedures of the CTD laboratory. This is a referral laboratory for parasitic infections in Italy, where slides for quality control of laboratories of different Italian regions are prepared. Although no specific quality assessment of microscopy for the study purpose was performed (as is obvious in a retrospective study), quality control, including intra- and inter-observer reproducibility, is systematically carried out according to standardized, certified procedures.

#### Treatment and prophylaxis

All patients were treated with a single anti-malarial, as for an acute malaria, once the diagnosis was established. The standard regimen followed at CTD was with oral quinine, 10 mg/kg × 3 for 3 days, followed by pyrimethamine–sulfametopyrazine (Metakelfin^®^) single dose [10], until the year 2002, then with artemisinin-based combination therapy (ACT), regimen currently in use. No further treatment or prophylaxis was indicated for patients remaining in Italy, while for those returning to endemic countries, a prophylaxis was indicated with doxycycline, and with mefloquine as a second choice. As a possible alternative for those not wishing to take a prophylaxis for a long time, an intermittent treatment was advised, empirically suggesting a periodicity of at least a full anti-malarial course every 3 months during the low-transmission season, and every month in the high-transmission season. Patients were advised to follow the first-line regimen for uncomplicated malaria of the host country.

### Statistical analysis

STATA IC 14 (StataCorp, 4905 Lakeway, College Station, TX, USA) was used for data analysis. For categorical variables the absolute and relative frequencies were calculated. For continuous variables, the median and IQ range were considered. The association between categorical variables was evaluated using the χ^2^ test. The Fisher exact test was used if appropriate. Uncertainty was quantified using a significance value of 0.05. Logistic regression was performed to study the potential association of the independent variables (outcomes) with the potential independent predictors.

### Ethical issues

Data were entered anonymously in the database. The competent Ethics Committee (*Comitato Etico delle province di Verona e Rovigo*) acknowledged the study protocol and formally authorized the study in September 2014 (protocol n. 43713).

## Results

### The study population

**Fig. 1:**
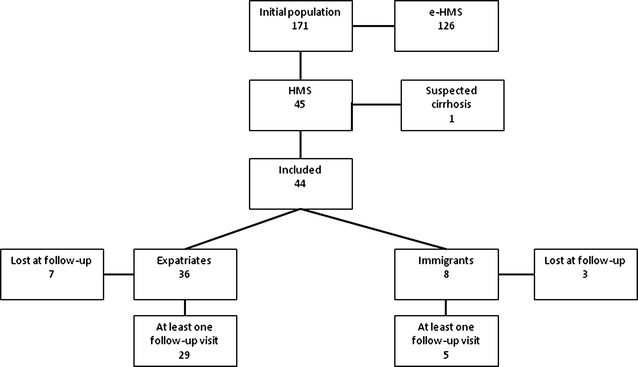
Patient flow diagram

Figure 1 shows the patient flow. From the database of 171 patients with splenomegaly and high anti-malarial antibody titre, 126 were classified as e-HMS [8], and were excluded from the analysis. One record, initially classified as a full-blown HMS, was then discarded as a thorough scrutiny of the patient clinical data raised the suspicion of a previously unrecognized liver cirrhosis. The remained 44 records were analysed.

Thirty-six patients were expatriates from non-endemic countries (one Mexican and 35 Italian) and eight were African immigrants. Seven expatriates and three immigrants were not seen again, while 29 and five, respectively, had at least one follow-up visit. Several patients had more than one follow-up visit, coinciding with periodical returns to Italy.

At least one short-term follow-up visit (≤6 months) before any further exposure to malaria, was available for half of the patients of both groups (18/36 expatriates, 4/8 immigrants). At least one long-term follow-up visit (>6 months) was available for 23/36 expatriates (of whom 22 had been re-exposed to malaria), and for only one immigrant (with an unclear history of re-exposure). The main symptoms, signs and laboratory findings of expatriate and immigrant patients at their first contact are summarized in Table 1. The spleen measure was based on ultrasound for all but three patients, for whom an estimation based on physical examination was done (not included in Table 1).

**Table 1:** Main characteristics of subjects diagnosed with HMS, immigrants and expatriates

Characteristic	All (n = 44)	Expatriates (n = 36)	Immigrants (n = 8)	p value
Age years; median (IQ range)	56.1 (15.8)	58.6 (10.0)	27.4 (14.2)	0.0001
Gender (F/M)	15/29	10/26	5/3	0.099
Exposure, years; median (IQ range)	20.0 (13.0)	20.0 (12.0)	27.5 (12.0)	0.2278
Presence of symptoms; ratio Y/N^a^	42/1	34/1	8/0	1.000
Fever; ratio Y/N*	24/19	19/16	5/3	1.000
Asthenia; ratio Y/N*	19/24	18/17	1/7	0.059
Left upper q. pain; ratio Y/N^a^	9/34	6/29	3/5	0.332
Intestinal discomfort ratio Y/N^a^	12/31	11/24	1/7	0.407
Spleen diameter in cm; median (IQ range)	18.0 (3.3)	18.0 (3.3)	17.6 (3.5)	0.5643
Hepatomegaly; ratio Y/N^a^	23/20	21/14	2/6	0.118
Hb, mg/dL normal 12–16; median (IQ range)	10.0 (3.0)	9.6 (2.2)	11.6 (4.0)	0.1695
RBC × 10^6^/µL, n 4.2–5.4; median (IQ range)	3.4 (1.0)	3.3 (0.9)	4.0 (1.1)	0.0239
Plt × 10^3^/µL, n 130–400; median (IQ range)	138.0 (81.0)	128.0 (76.0)	178.5 (100.0)	0.0702
ESR mm/h, n < 25; median (IQ range)	57.5 (43.5)	59.0 (38.0)	51.0 (86.0)	0.7352
IgM, g/L, n < 2.5; median (IQ range)	6.4 (3.9)	6.8 (3.8)	5.0 (12.6)	0.9738
Plasmodium in blood; ratio Y/N^b^	22/16	17/15	5/1	0.370
Improved at T1; ratio Y/N	20/2	16/2	4/0	1.000

^a^Info on symptoms lacking for one patient

^b^Malaria search lacking for six patients

Most of the subjects were symptomatic at presentation (Table 1). Fever (usually low-grade), asthenia, intestinal discomfort and pain at the left upper abdominal quadrant were the most frequent symptoms recorded. No significant difference was found between the two main ethnic groups for most of the symptoms. Asthenia was reported by more than half of the expatriates (18/35 or 51 %) versus only 1/7 immigrants (14 %) (p = 0.059). Hepatomegaly (usually moderate) was found in 23/43 subjects (53 %), 21/35 expatriates (67 %) and 2/8 immigrants (25 %), respectively (p = 0.118). For one subject (expatriate) the data on symptoms and signs were incomplete.

### Parasite detection

A search for malaria parasites was carried out for 32/36 Italian subjects (89 %), and 6/8 immigrants (75 %), with a positive result in 17/32 (53 %) and 5/6 (71 %), respectively (p = 0.370). The parasite density was low or very low in most cases (Table 2). Of the 38 cases for which data were recorded, 16 (42 %) resulted negative, nine (24 %) were positive at QBC only, and nine more had a parasite density (as quantified at thick film) below 500 parasites/μL. Only one case had a parasite density higher than 2000 parasites/μL. Of the 38 subjects with a malaria search carried out, fever was present in 7/15 (47 %) of those with a negative result and in 16/23 (70 %) of those with a positive result (p = 0.283). In particular, fever was present in all the four subjects with a parasite density >500/μL, while it was lacking in 7/19 (37 %) of those with a lower parasite density and in 5/9 (56 %) of those with parasites found at QBC only.

**Table 2:** Malaria parasites in blood

Parasitaemia (N/µL)	All	Expatriates	Immigrants
Negative	16 (42 %)	15 (47 %)	1 (17 %)
QBC^a^	9 (24 %)	7 (22 %)	2 (33 %)
1–500/µL	9 (24 %)	6 (19 %)	3 (50 %)
501–2000/µL	3 (8 %)	3 (9 %)	0 (0 %)
>2000/µL	1 (2 %)	1 (3 %)	0 (0 %)
Total	38	32	6

p = 0.410, Fisher’s exact

^a^Negative blood films, parasites found at QBC only

### Short-term outcome

**Fig. 2:**
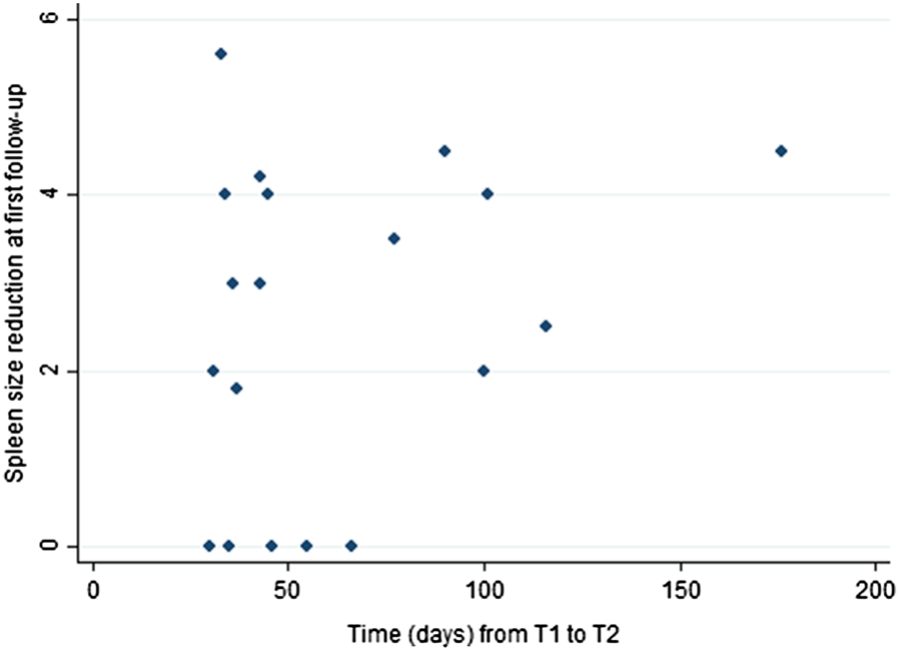
The reduction of the spleen size over time (short-term outcome)

Twenty-four subjects had a short-term follow-up visit (<6 months after treatment and with no re-exposure in between). The median time elapsed between initial and follow-up visit was 45.5 days (IQ range 66). One subject was impossible to classify as the spleen diameter was unchanged and the IgM value at follow-up was not available, while for another subject there was no recorded assessment of the spleen measure at follow-up. The results of the remaining 22 subjects are reported in Table 3. Most had improved, and none had worsened. The reduction of the spleen size over time (as recorded for all the subjects who had an echography measurement available at baseline and follow-up) is plotted in Fig. 2.

**Table 3:** Outcome at the first follow-up visit for subjects not re-exposed to malaria (N. 22), according to nationality

Outcome	Expatriates	Immigrants	Total
Cured or improved	16	4	20
Unchanged	2	0	2
Total	18	4	22

p = 1.000, Fisher’s exact

### Long-term outcome

Twenty-four subjects had at least one long-term follow-up visit and a reliable history concerning re-exposure or not to malaria. The median time elapsed between the initial and the last visit was 4.12 years (IQ range 2.77). Only two subjects had not been re-exposed. One was a 27 years old immigrant woman from Ghana, first observed in March 1993, who never returned to her country and was last seen after 3 years, when she was perfectly cured (with an initial spleen longitudinal diameter of 17 cm and a final one of 11 cm). The other was an expatriate missionary sister aged 63 when she was first seen in March 2001, with a huge spleen (24 cm, described as occupying most of the abdomen), showing nevertheless a significant improvement over time, with a 6-cm reduction of the longitudinal spleen diameter and a normalization of IgM when she was last seen in April 2004. Both had received a standard anti-malarial treatment at the first encounter, with no further prophylaxis. The results of the remaining 22 patients are summarized in Table 4. All were expatriates. Of the nine subjects reporting having followed the recommended anti-malarial prophylaxis (usually doxycycline), eight were improved or cured, and so were 10/13 subjects not reporting a prophylaxis, but an intermittent treatment instead (p = 0.616). Of the independent potential predictors included in the logistic regression model (including age, gender, symptoms, and in particular fever, presence of malaria parasites in blood, prophylaxis) none was significantly associated with the short-term or long-term outcome.

**Table 4:** Outcome at the last follow-up visit for subjects re-exposed to malaria (N. 22), according to the reported prophylaxis or intermittent treatment

Outcome	Prophylaxis	Intermittent treatment	**Total**
Cured or improved	8	10	18
Unchanged or progressed	1	3	4
Total	9	13	22

p = 0.616, Fisher’s exact

### Case description

The main findings of two particularly informative cases are briefly outlined below.

#### Case 1

Italian, female, non-governmental organization, volunteer in Cameroon, consulted for the first time in November 1996, when 57 years old. She had lived in the African country for 22 years. She presented with low-grade fever and a profound asthenia. The liver was moderately enlarged, while the spleen longitudinal diameter was 18 cm (she was 155 cm high). The laboratory showed marked anaemia (red blood cells (RBC), 2.67 × 10^6^/μL, Haemoglobin (Hb) 7.7 g/L) and leucopaenia (white blood cells (WBC), 2.17 × 10^3^/μL), a moderately low platelet count (PLT, 92 × 10^3^/μL), raised lactic-dehydrogenase (LDH) (747 U/L, N < 250), and IgM (4.7 g/L, N < 2.5). Both the thick and thin film for malaria were negative, but the QBC showed very scanty trophozoites, compatible with *P. falciparum*. The anti-malarial antibody titre (IFAT, BioMérieux) was ≥160 (not further diluted). She was treated with quinine, 10 mg/kg × 3/day for 3 days, followed by a single dose of pyrimethamine–sulfametoxazole, the standard regimen for acute malaria at that time at CTD [10]. A short-term follow-up was not possible as the patient insisted on returning to the host country for urgent matters, but she agreed to follow a strict prophylaxis with doxycycline and to return for a follow-up as early as possible. In fact, she represented after almost 1 year, in November 1997. She reported having been compliant with the prophylaxis advised and feeling much better. The spleen diameter had decreased to 13 cm, the main laboratory findings had greatly improved, and the IgM were now normal (1.4 g/L). However, in consideration of the previous findings, she was advised not to return to Cameroon, but she did not follow the advice and presented again at CTD 2½ years later (April 2000). This time she had not followed any prophylaxis, nor the alternative advice of an intermittent treatment, due to concern for toxicity. She showed a marked progression, with similar symptoms as at presentation, a spleen diameter of 19 cm, IgM 5.6, and other laboratory findings similar to the first visit, but for a negative malaria thick film and QBC. She was treated for malaria again, and returned to Cameroon despite contrary advice.

#### Case 2

Italian, male, Catholic missionary in Uganda for 27 years, 64 years old, consulted CTD in August 1998 for a medical check-up while he was on home leave. He reported a long history of acute malaria episodes, while in the last few years he said that he had often had malaria “without fever”. He identified the latter condition on the basis of “typical symptoms” (mainly a profound weakness), and took a short course of quinine when this occurred. The remaining clinical history was negative. He declared not having any particular complaint at presentation. The physical examination revealed a moderate hepatomegaly and a marked splenomegaly, with the lower pole well beyond the umbilical line and an echografic longitudinal diameter of 21 cm. The laboratory findings were similar to the previous case, with RBC 3.5 × 10^6^/μL, WBC 4.84 × 10^3^/μL, PLT 145 10^3^/μL, bilirubin 25.5 μmol/L, LDH 553 U/L, and IgM 9.2 g/L. Both the thick and thin film for malaria were negative, and so was the QBC. The anti-malarial antibody titre was 10,240. He was treated with the same regimen as Case 1. Shortly afterwards he had to go back to Uganda, therefore a short-term follow-up was not possible. An anti-malarial prophylaxis was recommended, either with doxycycline or with mefloquine (he had already taken the latter drug for treatment with no side effects). It was proposed the patient should come back to follow-up on his next return to Italy, initially planned in 1 year, considering that a long-term anti-malarial prophylaxis was not generally recommended because of potential toxicity. Nevertheless, he returned to medical observation after almost 5 years (July 2003), during which he reported having regularly taken one tablet of mefloquine once a week for the whole of the 5 years, and declared that he had “never been healthier”. The spleen diameter had decreased by 4 cm and the blood cell count, as well as the IgM value (0.33 g/L), were normal.

## Discussion

This paper describes the largest series ever published of HMS in a non-endemic country. The spleen measure required for case definition of HMS has been variable [4]. Most papers until recently have relied on physical examination. Many authors have reported following Fakunle’s criteria, requiring a spleen size bigger than 10 cm below the costal margin. Others based the spleen size assessment on Hackett’s criteria [11], usually requiring at least a Hackett’s Grade II (spleen palpable, but not beyond a horizontal line halfway between the costal margin and umbilicus). Only a few papers have used an ultrasound scan to measure the spleen length, with a minimal longitudinal diameter to define HMS ranging from 15 cm [5] to 18 cm [6]. In this paper, the minimal diameter required for the case definition of the full-blown HMS is 16 cm, although patients with a smaller spleen size have also shown a tendency to progression [8]. Moreover, virtually all studies, including this one, have not related the spleen measure to the patient’s size, which is obviously a limitation.

### The study population: symptoms, signs, laboratory findings

Most of the study subjects (36) were Italians, mostly missionary people, while there were eight immigrants. The only important difference between the two populations was the median age as immigrants are on average younger people, compared to expatriates who have spent decades in a foreign country. Asthenia and hypersplenism (with a lower median RBC and platelet count) were more common in expatriates, while the two populations were similar in all other respects (although the immigrant patient population was too small to detect less marked differences). Most patients were symptomatic at presentation, although symptoms were usually vague, similarly reported in literature [4]. Fever (almost invariably low-grade) was present in more than half of the subjects, and did not appear to be significantly correlated to the presence of malaria parasites in blood, although it was present in all the four patients with a parasite density over 500/μL. Parasitaemia was low or very low in all subjects, with only one case with >2000 parasites/μL (roughly corresponding to 0.05 %), and one quarter of the positive subjects having malaria parasites only identified at QBC. Although for positive patients an acute malaria in semi-immune subjects cannot be entirely excluded, the similarity in all other respects between positive and negative subjects, and the very low, average parasite density in the former, makes this hypothesis unlikely. The patient population appears to represent a continuum, with malaria parasites possibly present in most or all patients, but at a density too low to be detected for some of them.

### Outcome

All patients were treated with a single anti-malarial treatment, similar to acute malaria. In no case was a prolonged regimen at prophylactic dose administered, unless/until the patient eventually returned to a malaria-endemic country. Patients were always advised to avoid re-exposure and to choose a non-malarious country/area for residence if returning abroad, but this advice was rarely followed. In case of re-exposure, an effective anti-malarial prophylaxis (taking into account the drug-resistance profile of the country) was strongly recommended, as it was considered that the risk of progression of the syndrome outweighed that of the drug’s side effects. The short-term outcome of most of the subjects assessed after treatment, in the absence of re-exposure, was favourable, with a comparatively quick decrease of the spleen size (Fig. 2). The time elapsed between treatment and short-term follow-up was generally quite short (6 weeks on average), although identical to the largest series published so far in a non-endemic country [6]. In studies carried out in endemic countries, the follow-up time has been very variable, ranging from 1 month to 2 years [4]. With a longer interval, possibly a higher proportion of patients would have been completely cured at follow-up. On the other hand, anecdotal reports indicate that patients with HMS and no re-exposure could improve or heal, even without treatment [12]. It may well be possible that this would have occurred in the patients of the present series, provided that they remained unexposed for a sufficient time to spontaneously clear malaria parasites. The outcome was also favourable for most of the subjects seen again after a variable period of time of re-exposure to malaria (Table 4). All but one of those who declared having adhered to the recommended prophylaxis were cured or had improved, and so were most of those who had not adhered to prophylaxis, but had followed the alternative recommendation of an intermittent treatment. Case 1, however, suggests that re-exposure, in the absence of prophylaxis or intermittent treatment, is likely to trigger the relapse of the syndrome in a much shorter time than the long exposure to malaria usually required to develop HMS. On the contrary, Case 2 avoided any re-infection with a very long-term prophylaxis with mefloquine (declaring no side effects) and did not have any relapse. In the other cases, the prophylactic regimen used was with doxycycline.

### Chronic malaria and HMS

The term ‘chronic malaria’ has usually been used to define asymptomatic or pauci-symptomatic carriers of malaria parasites in blood [13–15]. However, it has recently been argued that asymptomatic malaria does not exist, and the presence of Plasmodia in blood, regardless of the presence or absence of acute symptoms and of fever in particular, should be sufficient to define chronic malaria as a disease warranting treatment [2]. HMS can be considered the most severe form of chronic malaria, although often with negative malaria blood tests. Moreover, the risk of evolution of a milder splenomegaly (with or without malaria parasites found in blood), defined as early-HMS, to a full-blown HMS has recently been suggested [8]. Splenomegaly (of any size) has been considered as a proxy for malaria parasitaemia for years and the ‘spleen rate’ has been used as a surrogate for malaria prevalence surveys [16]. If it is accepted that patients with chronic malaria should be treated, then splenomegalic patients in malaria-endemic areas should also be presumptively treated, even in the absence of a positive malaria microscopy or RDT.

### How to treat chronic malaria and HMS

Evidence in the past few years [4] and from the present series suggests that the constant presence of malaria parasites in blood is necessary to trigger, as well as to sustain, the syndrome, contrary to the traditional view that HMS, once established, does not require the presence of *Plasmodium* and tends to evolve, if untreated, similar to an auto-immune disease. According to the traditional view, long-term use of chloroquine works as an immune-modulating drug, rather than an anti-malarial [7]. In this series, all patients were treated with a single anti-malarial treatment, as for an acute malaria attack, and regardless the result of malaria search. No long-term prophylaxis was administered to those who did not return to endemic countries. For those patients who had planned to return to the host, malaria-endemic country, the prophylactic regimen advised was doxycycline, with mefloquine as a second choice. Chloroquine was never advised, due to the widespread resistance to this drug. It seems clear then that the apparent response to prophylaxis observed in most patients was due to the prevention of malaria infection, and not to ‘immune modulation’. However, a long-term (or even life-long) prophylaxis is practically not feasible in endemic countries. An intermittent anti-malarial treatment would be a logical alternative, as it has been already extensively used for some at-risk categories such as pregnant women and children [17, 18].

### Study limitations

This study was retrospective, and for this reason follow-up data are incomplete and very variable in time. A recall bias is likely to have occurred, and therefore the details of prophylaxis or intermittent treatment should be taken with caution. A selection bias cannot be excluded either and some patients, especially of the first years, may have been missed. The potential problems of the case definition have been discussed above. PCR for malaria is not used at CTD for routine diagnosis, therefore this information is lacking for our patients. It is possible that some of the cases with negative blood films and QBC would have been found positive by the more sensitive PCR. It has recently been suggested by McGregor et al. [5] that subjects with a positive PCR would be more likely to respond to therapy than negative subjects, indicating a role of molecular diagnosis in the case definition of the syndrome. In their series of seven patients, only three subjects that did not respond to therapy had negative blood films and PCR, seemingly indicating that the syndrome requires the presence of *Plasmodium* in blood. So the Authors suggest that a negative PCR predicts a treatment failure. On the other hand, in the present and much larger series most of the patients improved at follow-up, and among those who didn’t, some had positive malaria films or QBC.

## Conclusion

The hyper-reactive malarial splenomegaly (HMS) is the most severe form of chronic malaria, and not a different disease. A single anti-malarial treatment is sufficient to cure/improve most cases. However, reinfection must be prevented or immediately treated. As a lifelong prophylaxis does not appear to be feasible in malaria-endemic countries, considering the high number of chronic malaria carriers and patients with splenomegaly, research is needed in order to evaluate the efficacy of intermittent treatment as well as its best periodicity.
